# Crystal structure of tetra­kis­(imidazolium) hexa­kis­(imidazole-κ*N*)cobalt(II) bis­(benzene-1,3,5-tri­carboxyl­ate) dihydrate

**DOI:** 10.1107/S2056989026001660

**Published:** 2026-02-20

**Authors:** Jose de Jesus Velazquez Garcia, Edwige Nadia Pujol, Faegheh Khademhir, Bassima Knjo, Aliyenur Ekineken, Fabienne Hain, Simone Techert

**Affiliations:** a Deutsches Elektronen-Synchrotron DESY Notkestr 85 22607 Hamburg Germany; b Faculty of Science and Engineering, Sorbonne Université, 4 Place Jussieu, 75005, Paris, France; c BS 06 Berufliche Schule Chemie, Biologie, Pharmazie, Agrarwirtschaft, Ladenbeker Furtweg 151, 21033 Hamburg, Germany; d Institut für Röntgenphysik, Georg-August-Universität Göttingen, Friedrich-Hund-Platz 1, 37077 Göttingen, Germany; University of Durham, United Kingdom

**Keywords:** crystal structure, imidazol-1-ium, hexa­kis­(imidazole)­cobalt, benzene-1,3,5-tri­carboxyl­ate

## Abstract

The title compound, (C_3_H_5_N_2_)_4_[Co(C_3_H_4_N_2_)_6_](C_9_H_3_O_6_)_2_·2H_2_O (**1**), was synthesized by slow evaporation of mixed ethano­lic solutions of CoCl_2_, benzene-1,3,5-tri­carb­oxy­lic acid (H_3_btc) and imidazole (Im) at room temperature. The crystal structure comprises [Co(Im)_6_]^2+^ cations, btc^3−^ anions, Im^+^ cations and water mol­ecules in a 1:2:4:2 ratio. The crystal packing shows alternating layers stacked along the *c-*axis direction, linked primarily by hydrogen bonds of the types N—H⋯O (between cations and anions) and O—H⋯O (between anions and water mol­ecules).

## Abstract 2

The structure of a hexa­kis­(imidazole)­cobalt(II) bis­(benzene-1,3,5-tri­carboxyl­ate) tetra­(imidazole-1-ium) dihydrate compound was determined by single-crystal X-ray diffraction.

## 1. Chemical context

Rigid benzene di-, tri, and tetra-carb­oxy­lic acid, azolate-based ligands, as well as their derivatives are commonly employed as organic building blocks in the synthesis of metal–organic frameworks (MOFs) (Lin *et al.*, 2014 ▸). For example, benzene-1,3,5-tri­carb­oxy­lic acid (trimesic acid, H_3_btc) serves as a precursor in the synthesis of the well-known MOFs MIL-100 (Férey *et al.*, 2004 ▸) and HKUST-1 (Chui *et al.*, 1999 ▸). Azolate-based ligands, such as imidazole (Im) and 2-methyl­imidazole (2mIm), are key ligands in the synthesis of the zeolitic imidazolate frameworks (ZIFs), such as ZIF-4 and ZIF-8 (Park *et al.*, 2006 ▸). Over the last few years, we have used H_3_btc and 2mIm to synthesize a small coordination complex (de Velazquez-Garcia & Techert, 2022 ▸), various organic salts (Baletska *et al.*, 2023 ▸; Asprilla-Herrera *et al.*, 2025 ▸; Łukaszczyk *et al.*, 2025 ▸) and two mixed-ligand MOFs (Velazquez Garcia *et al.*, 2025 ▸). In this work, we used H_3_btc and Im to synthesize the title compound (**1**).
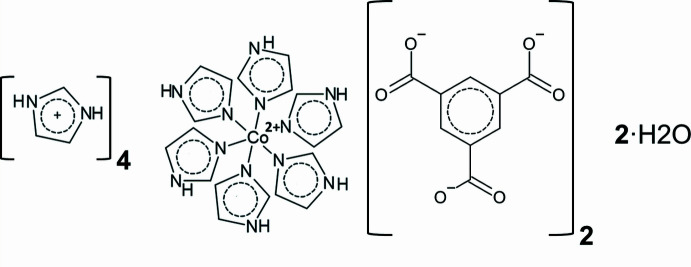


## 2. Structural commentary

**Figure 1:**
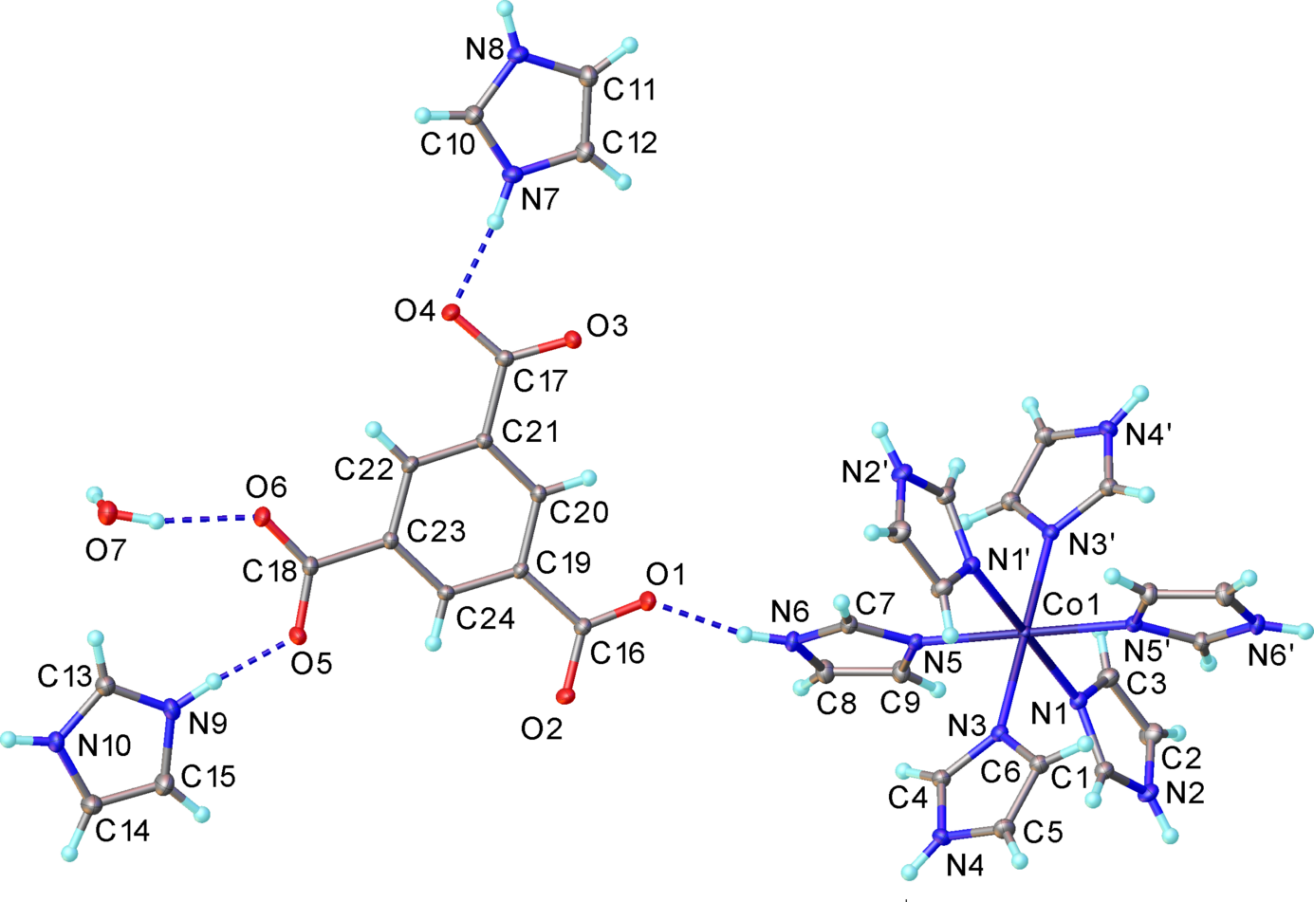
The mol­ecular structure of **1** with displacement ellipsoids drawn at the 50% probability level. Primed atoms are generated by the inversion operation 2 − *x*, −*y*, 1 − *z*

Compound **1** (Fig. 1 ▸) crystallizes in space group *P*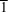. The complete formula unit comprises one hexa­kis­(imidazole)­cobalt(II) cation, four Im^+^ cations, two fully deprotonated btc^3−^ anions and two water mol­ecules. The asymmetric unit comprises one half of the formula unit (*Z*′ = 0.5) with the Co-containing cation lying about an inversion centre (Fig. 1 ▸). The Co—N bond lengths range from 2.1408 (10) to 2.1660 (10) Å.

The distortion from the ideal octa­hedral geometry of the Co-containing cation was qu­anti­fied using the parameters Σ (Halcrow, 2011 ▸) and Θ (Marchivie *et al.*, 2005 ▸), obtained via the *OctaDist* program (Ketkaew *et al.*, 2021 ▸). While Σ summarizes the deviation of the N—Co—N angles from 90°, Θ indicates the degree of twist from a perfect octa­hedron towards a trigonal prism. Both parameters are equal to zero in an ideal octa­hedron. The calculated values of the distortion parameters Σ and Θ for Co1 are equal to 12 and 36°, respectively. Both parameters indicate a slight distortion of the coordination environment of the metal centre.

## 3. Supra­molecular features

**Figure 2:**
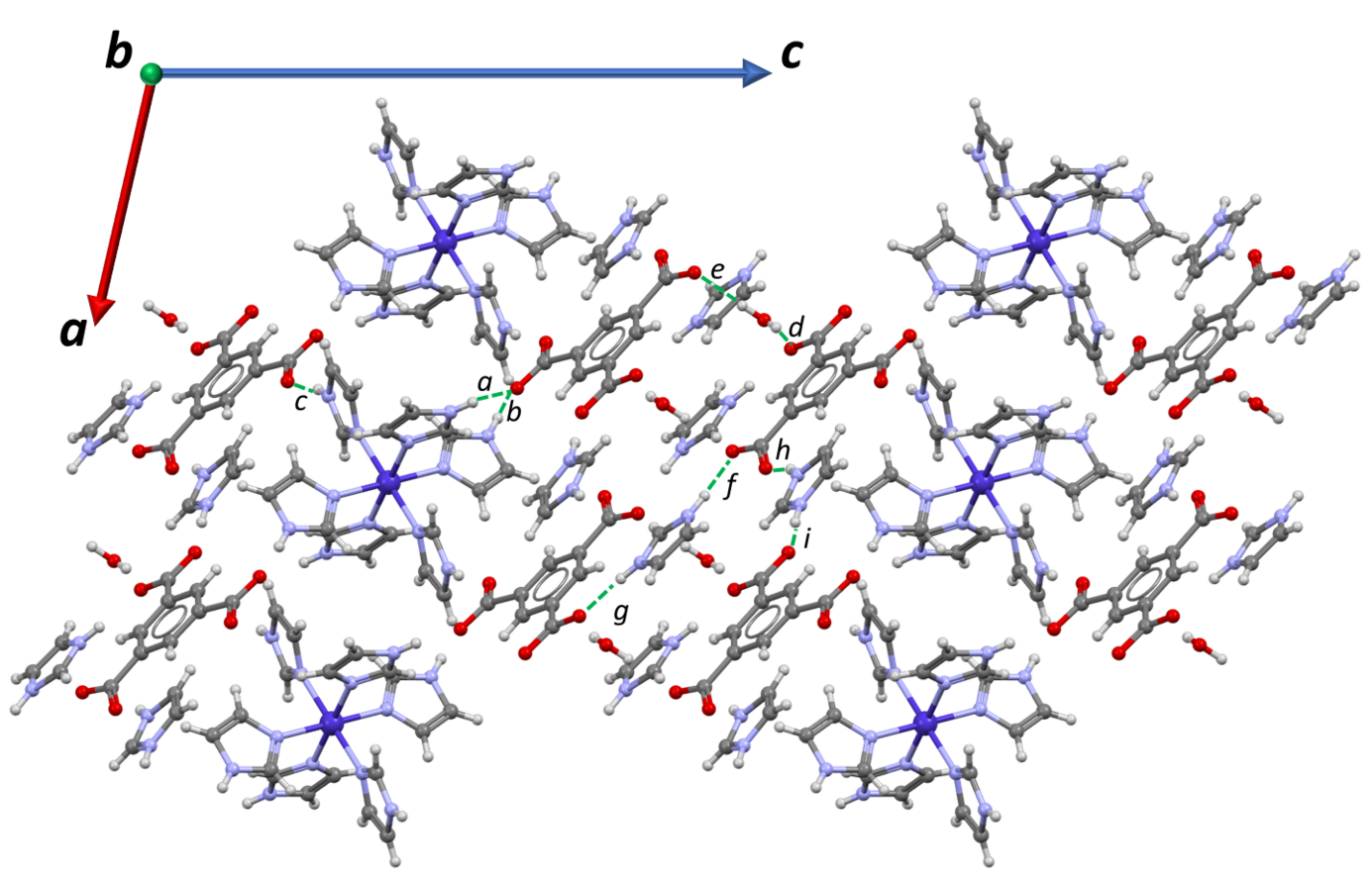
Packing diagram of **1** viewed down the *b* axis.

A packing diagram of the compound as viewed down the *b* axis is shown in Fig. 2 ▸. The figure shows a layered arrangement with all layers parallel to the *ab* plane. Three types of layers are observed: Plane A, formed by HIm^+^ cations and btc^3−^ anions; Plane *B*, consisting of hexa­kis­(imidazole)­cobalt(II) cations; and Plane *C*, composed of HIm^+^ cations and water mol­ecules. These layers stack in a repeating *A*–*B*–*A*–*C* sequence along the *c-*axis direction. Each layer inter­acts with others *via* hydrogen bonding of the N—H⋯O and O—H⋯O types. A summary of the hydrogen-bonding inter­actions is given in Table 1 ▸, showing that all possible donor and acceptor groups are involved in moderately strong hydrogen bonds. The latter form distinct patterns determined by graph-set analysis (Etter *et al.*, 1990 ▸; Bernstein *et al.*, 1995 ▸), which shows that structure **1** features only 12 discrete motifs.

## 4. Database survey

No reported structures of the title compound were found in the Cambridge Structural Database (CSD version 5.45, update of November 2023; Groom *et al.*, 2016 ▸). Some structures containing the hexa­kis­(imidazole)­cobalt(II) cation and polycarboxyl­ate anions were reported under the refcodes AGAXIS (Jyai & Srinivasan, 2019 ▸), BOVMIJ (Nie *et al.*, 2009 ▸) and EFIVOE (Tong *et al.*, 2002 ▸). However, none of them include btc^3−^ as anion.

## 5. Synthesis and crystallization

In a 4 mL vial, 100 µL of a 0.11 *M* ethano­lic solution of cobalt CoCl_2_·6H_2_O was mixed with 120 µL of a 1.58 *M* ethano­lic solution of Im. Then, 100 µL of a 0.12 *M* ethano­lic solution of H_3_btc was added to the mixture. The resulting mixture was gently shaken and allowed to evaporate slowly at room temperature. After three weeks, crystals of **1** were obtained.

## 6. Refinement

Crystal data, data collection and structure refinement details are summarized in Table 2 ▸. Imidazole H atoms were refined using a riding model with variable C—H or N—H distances and *U*_iso_(H) = 1.2*U*_eq_(C or N), water H atoms were refined with DFIX 0.87 and DANG 1.38 restraints and *U*_iso_(H) = 1.5*U*_eq_(O).

## Supplementary Material

Crystal structure: contains datablock(s) I. DOI: 10.1107/S2056989026001660/zv2041sup1.cif

Structure factors: contains datablock(s) I. DOI: 10.1107/S2056989026001660/zv2041Isup2.hkl

CCDC reference: 2531427

Additional supporting information:  crystallographic information; 3D view; checkCIF report

Additional supporting information:  crystallographic information; 3D view; checkCIF report

## Figures and Tables

**Table 1:** Hydrogen-bond geometry (Å,°)

*D*—H⋯*A*	Type	Graph-set	*D*—H	H⋯*A*	*D*⋯*A*	*D*—H⋯*A*
N2—H2⋯O2^i^	*a*	*D*, *D*^2^_2_(11)	0.860 (11)	1.888 (11)	2.7294 (15)	165.5 (7)
N4—H4⋯O2^ii^	*b*	*D*, *D*^2^_2_(11)	0.854 (12)	1.898 (11)	2.7336 (15)	165.9 (9)
N6—H6⋯O1	*c*	*D*, *D*^2^_2_(11)	0.872 (12)	1.834 (12)	2.6949 (15)	168.9 (7)
O7—H7*B*⋯O6	*d*	*D*	0.875 (18)	1.894 (18)	2.7558 (15)	167.9 (17)
O7—H7*C*⋯O4^iii^	*e*	*D*	0.900 (15)	1.947 (16)	2.8456 (15)	175.3 (16)
N7—H7*A*⋯O4	*f*	*D*	0.892 (13)	1.806 (14)	2.6932 (16)	172.6 (8)
N8—H8*A*⋯O6^v^	*g*	*D*	0.880 (10)	1.861 (10)	2.7350 (15)	172.1 (8)
N10—H10*A*⋯O3^iv^	*h*	*D*	0.881 (14)	1.791 (14)	2.6492 (15)	164.2 (5)
N9—H9*A*⋯O5	*i*	*D*	0.889 (12)	1.706 (12)	2.6031 (16)	174.6 (10)

Symmetry codes: (1) 1 − *x*, −*y*, 1 − *z*; (ii) 1 − *x*, 1 − *y*, 1 − *z*; (iii) 2 − *x*, 2 − *y*, −*z*; (iv) 1 + *x*, 1 + *y*, *z*; (v) 1 − *x*, 2 − *y*, −*z*.

**Table 2:** Experimental details

Crystal data	
Chemical formula	(C_3_H_5_N_2_)_4_[Co(C_3_H_4_N_2_)_6_](C_9_H_3_O_6_)_2_·2H_2_O
*M*_r_	1194.04
Crystal system, space group	Triclinic, *P* 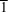
Temperature (K)	100
*a*, *b*, *c* (Å)	8.2752 (4), 8.8586 (4), 19.4212 (8)
α, β, γ (°)	95.310 (2), 101.624 (2), 102.854 (2)
*V* (Å^3^)	1345.42 (11)
*Z*	1
Radiation type	Mo *K*α
μ (mm^−1^)	0.41
Crystal size (mm)	1.0 × 0.6 × 0.3

Data collection	
Diffractometer	Bruker APEXII CCD area detector
Absorption correction	Multi-scan (*SADABS*; Krause *et al.*, 2015 ▸)
*T*_min_, *T*_max_	0.696, 0.746
No. of measured, independent and observed [*I* > 2σ(*I*)] reflections	41855, 6749, 6210
*R*_int_	0.027
(sin θ/λ)_max_ (Å^−1^)	0.670

Refinement	
*R*[*F*^2^ > 2σ(*F*^2^)], *wR*(*F*^2^), *S*	0.030, 0.078, 1.05
No. of reflections	6749
No. of parameters	407
No. of restraints	3
H-atom treatment	H atoms treated by a mixture of independent and constrained refinement
Δρ_max_, Δρ_min_ (e Å^−3^)	0.43, −0.36

Computer programs: *APEX2* (and *SAINT* (Bruker, 2016 ▸), *SHELXT2018/2* (Sheldrick, 2015*a* ▸), *SHELXL2018/3* (Sheldrick, 2015*b* ▸) and *OLEX2* (Dolomanov *et al.*, 2009 ▸).

## supplementary crystallographic information

### Tetrakis(imidazolium) hexakis(imidazole-κN)cobalt(II) bis(benzene-1,3,5-tricarboxylate) dihydrate Crystal data

**Table:**

(C_3_H_5_N_2_)_4_[Co(C_3_H_4_N_2_)_6_](C_9_H_3_O_6_)_2_·2H_2_O	*Z* = 1
*M**_r_* = 1194.04	*F*(000) = 621
Triclinic, *P*1	*D*_x_ = 1.474 Mg m^−^^3^
*a* = 8.2752 (4) Å	Mo *K*α radiation, λ = 0.71073 Å
*b* = 8.8586 (4) Å	Cell parameters from 9831 reflections
*c* = 19.4212 (8) Å	θ = 2.4–28.4°
α = 95.310 (2)°	µ = 0.41 mm^−^^1^
β = 101.624 (2)°	*T* = 100 K
γ = 102.854 (2)°	Irregular, red
*V* = 1345.42 (11) Å^3^	1.0 × 0.6 × 0.3 mm

### Tetrakis(imidazolium) hexakis(imidazole-κN)cobalt(II) bis(benzene-1,3,5-tricarboxylate) dihydrate Data collection

**Table:**

Bruker APEXII CCD area detector diffractometer	6210 reflections with *I* > 2σ(*I*)
phi and ω scans	*R*_int_ = 0.027
Absorption correction: multi-scan (SADABS; Krause *et al.*, 2015)	θ_max_ = 28.4°, θ_min_ = 2.2°
*T*_min_ = 0.696, *T*_max_ = 0.746	*h* = −11→11
41855 measured reflections	*k* = −11→11
6749 independent reflections	*l* = −26→25

### Tetrakis(imidazolium) hexakis(imidazole-κN)cobalt(II) bis(benzene-1,3,5-tricarboxylate) dihydrate Refinement

**Table:**

Refinement on *F*^2^	Primary atom site location: dual
Least-squares matrix: full	Hydrogen site location: mixed
*R*[*F*^2^ > 2σ(*F*^2^)] = 0.030	H atoms treated by a mixture of independent and constrained refinement
*wR*(*F*^2^) = 0.078	*w* = 1/[σ^2^(*F*_o_^2^) + (0.0329*P*)^2^ + 0.8481*P*] where *P* = (*F*_o_^2^ + 2*F*_c_^2^)/3
*S* = 1.05	(Δ/σ)_max_ = 0.001
6749 reflections	Δρ_max_ = 0.43 e Å^−^^3^
407 parameters	Δρ_min_ = −0.36 e Å^−^^3^
3 restraints	

### Tetrakis(imidazolium) hexakis(imidazole-κN)cobalt(II) bis(benzene-1,3,5-tricarboxylate) dihydrate Special details

**Table:**

Geometry. All esds (except the esd in the dihedral angle between two l.s. planes) are estimated using the full covariance matrix. The cell esds are taken into account individually in the estimation of esds in distances, angles and torsion angles; correlations between esds in cell parameters are only used when they are defined by crystal symmetry. An approximate (isotropic) treatment of cell esds is used for estimating esds involving l.s. planes.

### Tetrakis(imidazolium) hexakis(imidazole-κN)cobalt(II) bis(benzene-1,3,5-tricarboxylate) dihydrate Fractional atomic coordinates and isotropic or equivalent isotropic displacement parameters (Å^2^)

**Table:**

	*x*	*y*	*z*	*U*_iso_*/*U*_eq_	
Co1	1.000000	0.000000	0.500000	0.00894 (6)	
O2	0.39146 (12)	0.54791 (11)	0.32042 (5)	0.01739 (19)	
O3	0.94002 (12)	0.63711 (11)	0.12979 (5)	0.01620 (18)	
O4	0.87107 (12)	0.82054 (11)	0.06753 (5)	0.01915 (19)	
O5	0.29103 (12)	1.00222 (11)	0.20174 (5)	0.01881 (19)	
O6	0.44531 (12)	1.09291 (11)	0.12540 (5)	0.01906 (19)	
O1	0.58037 (13)	0.42621 (11)	0.28993 (5)	0.0215 (2)	
O7	0.32574 (14)	1.33180 (13)	0.06869 (6)	0.0242 (2)	
H7B	0.357 (2)	1.259 (2)	0.0918 (10)	0.036*	
H7C	0.266 (2)	1.279 (2)	0.0260 (8)	0.036*	
N5	0.79448 (13)	0.06066 (12)	0.42876 (5)	0.01174 (19)	
N1	0.83209 (13)	−0.21112 (12)	0.51648 (5)	0.01251 (19)	
N3	0.94168 (13)	0.12759 (12)	0.58700 (5)	0.01188 (19)	
N2	0.70821 (14)	−0.38463 (13)	0.57651 (6)	0.0165 (2)	
H2	0.6676 (8)	−0.4237 (8)	0.6102 (7)	0.020*	
N4	0.80835 (14)	0.25934 (12)	0.64879 (5)	0.0144 (2)	
H4	0.7336 (15)	0.3052 (9)	0.6586 (2)	0.017*	
N7	1.13972 (14)	0.77292 (13)	0.01793 (6)	0.0164 (2)	
H7A	1.0560 (17)	0.7887 (3)	0.0381 (4)	0.020*	
N6	0.64139 (13)	0.19585 (13)	0.36508 (6)	0.0149 (2)	
H6	0.6141 (6)	0.2733 (16)	0.3448 (4)	0.018*	
N10	−0.03117 (14)	1.38098 (13)	0.18656 (6)	0.0164 (2)	
H10A	−0.0542 (5)	1.4673 (18)	0.1727 (3)	0.020*	
N8	1.33265 (14)	0.80451 (13)	−0.04292 (6)	0.0164 (2)	
H8A	1.3993 (13)	0.8447 (8)	−0.0701 (5)	0.020*	
N9	0.09204 (14)	1.19201 (13)	0.20257 (6)	0.0173 (2)	
H9A	0.1651 (15)	1.1309 (12)	0.20117 (6)	0.021*	
C16	0.49883 (15)	0.52867 (14)	0.28451 (6)	0.0125 (2)	
C24	0.44916 (15)	0.75867 (14)	0.22124 (6)	0.0122 (2)	
H24	0.3629 (15)	0.76491 (18)	0.2442 (4)	0.015*	
C17	0.84952 (15)	0.73004 (14)	0.11361 (6)	0.0132 (2)	
C21	0.70671 (15)	0.73746 (14)	0.15060 (6)	0.0119 (2)	
C19	0.53436 (15)	0.64081 (14)	0.23185 (6)	0.0114 (2)	
C5	0.95059 (16)	0.24602 (15)	0.69538 (7)	0.0160 (2)	
H5	0.9847 (6)	0.2846 (7)	0.7439 (9)	0.019*	
C23	0.49277 (15)	0.86762 (14)	0.17627 (6)	0.0123 (2)	
C7	0.79417 (15)	0.19669 (14)	0.40538 (6)	0.0139 (2)	
H7	0.8902 (17)	0.2840 (15)	0.41584 (19)	0.017*	
C18	0.40284 (15)	0.99806 (14)	0.16688 (6)	0.0139 (2)	
C22	0.62126 (15)	0.85571 (14)	0.14128 (6)	0.0130 (2)	
H22	0.6507 (5)	0.9277 (13)	0.1112 (5)	0.016*	
C20	0.66138 (15)	0.63031 (14)	0.19548 (6)	0.0122 (2)	
H20	0.7174 (10)	0.5491 (14)	0.20144 (12)	0.015*	
C6	1.03247 (16)	0.16504 (14)	0.65676 (6)	0.0143 (2)	
H6A	1.1383 (18)	0.1379 (5)	0.6754 (3)	0.017*	
C8	0.53656 (16)	0.05072 (15)	0.36181 (7)	0.0167 (2)	
H8	0.426 (2)	0.0163 (7)	0.3381 (5)	0.020*	
C4	0.80784 (16)	0.18688 (14)	0.58473 (6)	0.0139 (2)	
H4A	0.7219 (15)	0.1792 (2)	0.5430 (7)	0.017*	
C9	0.63174 (16)	−0.03136 (15)	0.40138 (7)	0.0153 (2)	
H9	0.5925 (7)	−0.1351 (19)	0.40891 (15)	0.018*	
C1	0.78258 (15)	−0.23377 (15)	0.57635 (7)	0.0143 (2)	
H1	0.7975 (3)	−0.1552 (14)	0.6134 (7)	0.017*	
C11	1.33407 (17)	0.66799 (15)	−0.01452 (7)	0.0176 (2)	
H11	1.4046 (14)	0.6019 (13)	−0.02044 (13)	0.021*	
C2	0.70918 (17)	−0.46475 (15)	0.51278 (7)	0.0193 (3)	
H2A	0.6658 (8)	−0.573 (2)	0.4975 (3)	0.023*	
C14	−0.12181 (18)	1.28397 (16)	0.22441 (7)	0.0209 (3)	
H14	−0.2210 (19)	1.2975 (3)	0.2406 (3)	0.025*	
C3	0.78548 (16)	−0.35705 (15)	0.47609 (7)	0.0158 (2)	
H3	0.8036 (4)	−0.3788 (4)	0.4303 (8)	0.019*	
C13	0.09671 (17)	1.32276 (16)	0.17442 (7)	0.0180 (2)	
H13	0.1751 (15)	1.3660 (8)	0.1505 (5)	0.022*	
C12	1.21281 (17)	0.64859 (15)	0.02371 (7)	0.0179 (2)	
H12	1.1845 (6)	0.5667 (16)	0.0490 (5)	0.022*	
C15	−0.04403 (18)	1.16575 (17)	0.23437 (8)	0.0214 (3)	
H15	−0.0766 (7)	1.0822 (17)	0.2583 (5)	0.026*	
C10	1.21386 (17)	0.86566 (15)	−0.02249 (7)	0.0175 (2)	
H10	1.1871 (5)	0.9587 (18)	−0.0347 (2)	0.021*	

### Tetrakis(imidazolium) hexakis(imidazole-κN)cobalt(II) bis(benzene-1,3,5-tricarboxylate) dihydrate Atomic displacement parameters (Å^2^)

**Table:**

	*U*^11^	*U*^22^	*U*^33^	*U*^12^	*U*^13^	*U*^23^
Co1	0.01030 (11)	0.00904 (11)	0.00936 (10)	0.00363 (8)	0.00425 (8)	0.00344 (8)
O2	0.0221 (5)	0.0178 (4)	0.0205 (4)	0.0104 (4)	0.0140 (4)	0.0104 (4)
O3	0.0195 (4)	0.0167 (4)	0.0194 (4)	0.0109 (3)	0.0108 (4)	0.0083 (3)
O4	0.0234 (5)	0.0221 (5)	0.0223 (5)	0.0133 (4)	0.0152 (4)	0.0141 (4)
O5	0.0210 (5)	0.0209 (5)	0.0242 (5)	0.0140 (4)	0.0136 (4)	0.0124 (4)
O6	0.0228 (5)	0.0199 (5)	0.0235 (5)	0.0127 (4)	0.0128 (4)	0.0140 (4)
O1	0.0282 (5)	0.0215 (5)	0.0275 (5)	0.0170 (4)	0.0177 (4)	0.0164 (4)
O7	0.0294 (5)	0.0264 (5)	0.0259 (5)	0.0166 (4)	0.0119 (4)	0.0133 (4)
N5	0.0134 (5)	0.0128 (5)	0.0112 (4)	0.0052 (4)	0.0046 (4)	0.0035 (4)
N1	0.0130 (5)	0.0124 (5)	0.0138 (5)	0.0038 (4)	0.0048 (4)	0.0046 (4)
N3	0.0139 (5)	0.0111 (5)	0.0127 (5)	0.0040 (4)	0.0057 (4)	0.0042 (4)
N2	0.0190 (5)	0.0153 (5)	0.0190 (5)	0.0043 (4)	0.0101 (4)	0.0084 (4)
N4	0.0166 (5)	0.0154 (5)	0.0155 (5)	0.0082 (4)	0.0083 (4)	0.0039 (4)
N7	0.0176 (5)	0.0174 (5)	0.0183 (5)	0.0065 (4)	0.0098 (4)	0.0060 (4)
N6	0.0173 (5)	0.0155 (5)	0.0157 (5)	0.0083 (4)	0.0053 (4)	0.0075 (4)
N10	0.0210 (5)	0.0152 (5)	0.0180 (5)	0.0101 (4)	0.0077 (4)	0.0065 (4)
N8	0.0178 (5)	0.0175 (5)	0.0178 (5)	0.0057 (4)	0.0097 (4)	0.0069 (4)
N9	0.0193 (5)	0.0172 (5)	0.0200 (5)	0.0108 (4)	0.0067 (4)	0.0058 (4)
C16	0.0138 (5)	0.0117 (5)	0.0140 (5)	0.0041 (4)	0.0055 (4)	0.0046 (4)
C24	0.0132 (5)	0.0136 (5)	0.0127 (5)	0.0056 (4)	0.0063 (4)	0.0042 (4)
C17	0.0151 (5)	0.0135 (5)	0.0136 (5)	0.0054 (4)	0.0066 (4)	0.0036 (4)
C21	0.0130 (5)	0.0131 (5)	0.0120 (5)	0.0055 (4)	0.0053 (4)	0.0036 (4)
C19	0.0132 (5)	0.0111 (5)	0.0116 (5)	0.0040 (4)	0.0048 (4)	0.0043 (4)
C5	0.0183 (6)	0.0182 (6)	0.0125 (5)	0.0058 (5)	0.0043 (4)	0.0014 (4)
C23	0.0136 (5)	0.0128 (5)	0.0130 (5)	0.0059 (4)	0.0046 (4)	0.0043 (4)
C7	0.0147 (5)	0.0139 (6)	0.0157 (5)	0.0058 (4)	0.0056 (4)	0.0056 (4)
C18	0.0149 (5)	0.0144 (6)	0.0154 (5)	0.0068 (4)	0.0049 (4)	0.0059 (4)
C22	0.0161 (5)	0.0130 (5)	0.0132 (5)	0.0055 (4)	0.0067 (4)	0.0061 (4)
C20	0.0141 (5)	0.0115 (5)	0.0134 (5)	0.0056 (4)	0.0047 (4)	0.0039 (4)
C6	0.0155 (6)	0.0155 (6)	0.0133 (5)	0.0054 (4)	0.0046 (4)	0.0033 (4)
C8	0.0149 (6)	0.0160 (6)	0.0193 (6)	0.0052 (5)	0.0018 (5)	0.0038 (5)
C4	0.0159 (6)	0.0149 (6)	0.0137 (5)	0.0061 (4)	0.0059 (4)	0.0042 (4)
C9	0.0153 (6)	0.0127 (6)	0.0184 (6)	0.0042 (4)	0.0034 (4)	0.0042 (4)
C1	0.0149 (5)	0.0150 (6)	0.0154 (5)	0.0048 (4)	0.0062 (4)	0.0053 (4)
C11	0.0194 (6)	0.0159 (6)	0.0212 (6)	0.0075 (5)	0.0076 (5)	0.0061 (5)
C2	0.0238 (6)	0.0124 (6)	0.0228 (6)	0.0024 (5)	0.0096 (5)	0.0040 (5)
C14	0.0222 (6)	0.0227 (7)	0.0251 (7)	0.0109 (5)	0.0128 (5)	0.0104 (5)
C3	0.0193 (6)	0.0139 (6)	0.0153 (6)	0.0032 (5)	0.0066 (5)	0.0030 (4)
C13	0.0204 (6)	0.0188 (6)	0.0193 (6)	0.0092 (5)	0.0082 (5)	0.0065 (5)
C12	0.0208 (6)	0.0150 (6)	0.0210 (6)	0.0057 (5)	0.0079 (5)	0.0077 (5)
C15	0.0229 (7)	0.0205 (7)	0.0263 (7)	0.0091 (5)	0.0102 (5)	0.0117 (5)
C10	0.0207 (6)	0.0167 (6)	0.0202 (6)	0.0078 (5)	0.0102 (5)	0.0079 (5)

### Tetrakis(imidazolium) hexakis(imidazole-κN)cobalt(II) bis(benzene-1,3,5-tricarboxylate) dihydrate Geometric parameters (Å, º)

**Table:**

Co1—N5^i^	2.1660 (10)	N8—C10	1.3309 (16)
Co1—N5	2.1660 (10)	N9—H9A	0.899 (18)
Co1—N1^i^	2.1598 (10)	N9—C13	1.3220 (17)
Co1—N1	2.1598 (10)	N9—C15	1.3762 (17)
Co1—N3^i^	2.1408 (10)	C16—C19	1.5161 (15)
Co1—N3	2.1408 (10)	C24—H24	0.923 (16)
O2—C16	1.2649 (14)	C24—C19	1.3923 (16)
O3—C17	1.2509 (15)	C24—C23	1.3973 (16)
O4—C17	1.2684 (15)	C17—C21	1.5117 (15)
O5—C18	1.2565 (15)	C21—C22	1.3940 (16)
O6—C18	1.2596 (15)	C21—C20	1.3884 (16)
O1—C16	1.2457 (15)	C19—C20	1.3940 (15)
O7—H7B	0.875 (15)	C5—H5	0.935 (17)
O7—H7C	0.899 (14)	C5—C6	1.3630 (17)
N5—C7	1.3268 (16)	C23—C18	1.5129 (16)
N5—C9	1.3797 (16)	C23—C22	1.3898 (16)
N1—C1	1.3261 (15)	C7—H7	0.950 (16)
N1—C3	1.3798 (16)	C22—H22	0.930 (16)
N3—C6	1.3806 (15)	C20—H20	0.942 (16)
N3—C4	1.3221 (15)	C6—H6A	0.969 (16)
N2—H2	0.861 (17)	C8—H8	0.907 (17)
N2—C1	1.3418 (16)	C8—C9	1.3616 (17)
N2—C2	1.3709 (17)	C4—H4A	0.951 (16)
N4—H4	0.853 (17)	C9—H9	0.939 (17)
N4—C5	1.3677 (17)	C1—H1	0.923 (17)
N4—C4	1.3449 (15)	C11—H11	0.930 (18)
N7—H7A	0.891 (17)	C11—C12	1.3547 (17)
N7—C12	1.3724 (17)	C2—H2A	0.939 (18)
N7—C10	1.3255 (16)	C2—C3	1.3623 (17)
N6—H6	0.872 (17)	C14—H14	0.964 (18)
N6—C7	1.3443 (16)	C14—C15	1.3549 (19)
N6—C8	1.3696 (17)	C3—H3	0.940 (16)
N10—H10A	0.881 (18)	C13—H13	0.909 (17)
N10—C14	1.3761 (16)	C12—H12	0.928 (17)
N10—C13	1.3275 (16)	C15—H15	0.928 (18)
N8—H8A	0.880 (17)	C10—H10	0.939 (18)
N8—C11	1.3762 (16)		
			
N5—Co1—N5^i^	180.00 (5)	C24—C19—C20	119.02 (10)
N1—Co1—N5	91.95 (4)	C20—C19—C16	119.09 (10)
N1—Co1—N5^i^	88.05 (4)	N4—C5—H5	127.0
N1^i^—Co1—N5^i^	91.95 (4)	C6—C5—N4	106.05 (11)
N1^i^—Co1—N5	88.05 (4)	C6—C5—H5	127.0
N1^i^—Co1—N1	180.0	C24—C23—C18	120.25 (10)
N3^i^—Co1—N5	91.00 (4)	C22—C23—C24	119.09 (11)
N3^i^—Co1—N5^i^	89.00 (4)	C22—C23—C18	120.66 (10)
N3—Co1—N5	89.00 (4)	N5—C7—N6	111.70 (11)
N3—Co1—N5^i^	91.00 (4)	N5—C7—H7	124.1
N3^i^—Co1—N1^i^	89.89 (4)	N6—C7—H7	124.1
N3^i^—Co1—N1	90.12 (4)	O5—C18—O6	125.43 (11)
N3—Co1—N1^i^	90.11 (4)	O5—C18—C23	116.47 (10)
N3—Co1—N1	89.88 (4)	O6—C18—C23	118.10 (10)
N3—Co1—N3^i^	180.0	C21—C22—H22	119.5
H7B—O7—H7C	103.7 (17)	C23—C22—C21	121.00 (11)
C7—N5—Co1	127.69 (8)	C23—C22—H22	119.5
C7—N5—C9	105.05 (10)	C21—C20—C19	121.11 (11)
C9—N5—Co1	127.16 (8)	C21—C20—H20	119.4
C1—N1—Co1	125.01 (9)	C19—C20—H20	119.4
C1—N1—C3	105.20 (10)	N3—C6—H6A	125.2
C3—N1—Co1	128.38 (8)	C5—C6—N3	109.68 (11)
C6—N3—Co1	128.28 (8)	C5—C6—H6A	125.2
C4—N3—Co1	126.51 (8)	N6—C8—H8	126.9
C4—N3—C6	105.22 (10)	C9—C8—N6	106.17 (11)
C1—N2—H2	126.5	C9—C8—H8	126.9
C1—N2—C2	107.03 (10)	N3—C4—N4	111.62 (11)
C2—N2—H2	126.5	N3—C4—H4A	124.2
C5—N4—H4	126.3	N4—C4—H4A	124.2
C4—N4—H4	126.3	N5—C9—H9	125.1
C4—N4—C5	107.43 (10)	C8—C9—N5	109.80 (11)
C12—N7—H7A	125.6	C8—C9—H9	125.1
C10—N7—H7A	125.6	N1—C1—N2	111.87 (11)
C10—N7—C12	108.89 (11)	N1—C1—H1	124.1
C7—N6—H6	126.4	N2—C1—H1	124.1
C7—N6—C8	107.28 (10)	N8—C11—H11	126.6
C8—N6—H6	126.4	C12—C11—N8	106.76 (11)
C14—N10—H10A	125.7	C12—C11—H11	126.6
C13—N10—H10A	125.7	N2—C2—H2A	126.8
C13—N10—C14	108.63 (11)	C3—C2—N2	106.45 (11)
C11—N8—H8A	125.6	C3—C2—H2A	126.8
C10—N8—H8A	125.6	N10—C14—H14	126.8
C10—N8—C11	108.75 (10)	C15—C14—N10	106.46 (11)
C13—N9—H9A	126.0	C15—C14—H14	126.8
C13—N9—C15	108.09 (11)	N1—C3—H3	125.3
C15—N9—H9A	126.0	C2—C3—N1	109.45 (11)
O2—C16—C19	117.73 (10)	C2—C3—H3	125.3
O1—C16—O2	125.05 (11)	N10—C13—H13	125.4
O1—C16—C19	117.21 (10)	N9—C13—N10	109.25 (11)
C19—C24—H24	119.6	N9—C13—H13	125.4
C19—C24—C23	120.75 (11)	N7—C12—H12	126.4
C23—C24—H24	119.6	C11—C12—N7	107.12 (11)
O3—C17—O4	123.53 (11)	C11—C12—H12	126.4
O3—C17—C21	119.16 (10)	N9—C15—H15	126.2
O4—C17—C21	117.31 (10)	C14—C15—N9	107.58 (12)
C22—C21—C17	119.91 (10)	C14—C15—H15	126.2
C20—C21—C17	121.05 (11)	N7—C10—N8	108.48 (11)
C20—C21—C22	119.01 (11)	N7—C10—H10	125.8
C24—C19—C16	121.82 (10)	N8—C10—H10	125.8
			
Co1—N5—C7—N6	−176.37 (8)	C5—N4—C4—N3	−0.13 (14)
Co1—N5—C9—C8	176.64 (8)	C23—C24—C19—C16	175.32 (11)
Co1—N1—C1—N2	−167.16 (8)	C23—C24—C19—C20	−1.64 (18)
Co1—N1—C3—C2	166.60 (9)	C7—N5—C9—C8	0.21 (14)
Co1—N3—C6—C5	−179.36 (8)	C7—N6—C8—C9	0.39 (14)
Co1—N3—C4—N4	179.56 (8)	C18—C23—C22—C21	178.90 (11)
O2—C16—C19—C24	−1.86 (17)	C22—C21—C20—C19	−0.97 (18)
O2—C16—C19—C20	175.09 (11)	C22—C23—C18—O5	−178.40 (12)
O3—C17—C21—C22	170.38 (12)	C22—C23—C18—O6	1.51 (18)
O3—C17—C21—C20	−7.97 (18)	C20—C21—C22—C23	0.19 (18)
O4—C17—C21—C22	−9.28 (17)	C6—N3—C4—N4	−0.07 (14)
O4—C17—C21—C20	172.38 (12)	C8—N6—C7—N5	−0.27 (14)
O1—C16—C19—C24	179.43 (12)	C4—N3—C6—C5	0.26 (14)
O1—C16—C19—C20	−3.62 (17)	C4—N4—C5—C6	0.28 (14)
N2—C2—C3—N1	0.12 (15)	C9—N5—C7—N6	0.04 (13)
N4—C5—C6—N3	−0.34 (14)	C1—N1—C3—C2	−0.20 (14)
N6—C8—C9—N5	−0.38 (14)	C1—N2—C2—C3	0.00 (15)
N10—C14—C15—N9	−0.07 (16)	C11—N8—C10—N7	0.04 (15)
N8—C11—C12—N7	0.07 (15)	C2—N2—C1—N1	−0.14 (15)
C16—C19—C20—C21	−175.35 (11)	C14—N10—C13—N9	0.26 (16)
C24—C19—C20—C21	1.69 (18)	C3—N1—C1—N2	0.21 (14)
C24—C23—C18—O5	0.63 (17)	C13—N10—C14—C15	−0.11 (16)
C24—C23—C18—O6	−179.45 (12)	C13—N9—C15—C14	0.23 (16)
C24—C23—C22—C21	−0.15 (18)	C12—N7—C10—N8	0.01 (15)
C17—C21—C22—C23	−178.19 (11)	C15—N9—C13—N10	−0.30 (15)
C17—C21—C20—C19	177.39 (11)	C10—N7—C12—C11	−0.05 (15)
C19—C24—C23—C18	−178.17 (11)	C10—N8—C11—C12	−0.07 (15)
C19—C24—C23—C22	0.88 (18)		

Symmetry code: (i) −*x*+2, −*y*, −*z*+1.

### Tetrakis(imidazolium) hexakis(imidazole-κN)cobalt(II) bis(benzene-1,3,5-tricarboxylate) dihydrate Hydrogen-bond geometry (Å, º)

**Table:**

*D*—H···*A*	*D*—H	H···*A*	*D*···*A*	*D*—H···*A*
O7—H7*B*···O6	0.88 (2)	1.89 (2)	2.7558 (14)	168 (2)
O7—H7*C*···O4^ii^	0.90 (1)	1.95 (2)	2.8455 (15)	175 (2)
N2—H2···O2^iii^	0.86	1.89	2.7295 (14)	166
N4—H4···O2^iv^	0.85	1.90	2.7337 (14)	166
N7—H7*A*···O4	0.89	1.81	2.6931 (14)	173
N6—H6···O1	0.87	1.83	2.6948 (14)	169
N10—H10*A*···O3^v^	0.88	1.79	2.6492 (14)	164
N8—H8*A*···O6^vi^	0.88	1.86	2.7351 (14)	172
N9—H9*A*···O5	0.90	1.71	2.6030 (14)	175

Symmetry codes: (ii) −*x*+1, −*y*+2, −*z*; (iii) −*x*+1, −*y*, −*z*+1; (iv) −*x*+1, −*y*+1, −*z*+1; (v) *x*−1, *y*+1, *z*; (vi) −*x*+2, −*y*+2, −*z*.
